# The Institutional Worker

**Published:** 1913-09-06

**Authors:** 


THE
### INSTITUTIONAL WORKER
Being a Special Supplement to "The Hospital"

### OUR BUREAU OF INFORMATION.

### Rules for Correspondents.

1. Every letter must be accompanied by the coupon to be cut from the back cover (inside page) of The Hospital, current issue, and must contain the name and address of the correspondent with pseudonym for publication if desired. Replies by post cannot be given, save under exceptional circumstances at the Editor's discretion.

2. Letters from Approved Homes in reply to special needs published in the Bureau should state terms and full particulars, and be sent prepaid under cover to the Editor of the Bureau with name written across coupon for identification.

3. Proprietors of Homes which have not yet been entered on the List of Approved Homes, but have spare accommodation likely to suit special needs, are invited to write for an application form for registration. The fee for registration, which includes two announcements of the Home in the Bureau and other privileges, is 10s.

4. All communications to be addressed to the Editor of The Hospital, 28 Southampton Street, Strand, London, W.C., and marked "Bureau of Information."

### INSTITUTIONAL FACTS AND FIGURES.

### Gaps in Hospital Accounts.

The average secretary, rightly in one sense, dreads nothing more than the suggestion of any further change or reform in his accounts. The Uniform System has chiefly produced critics among men in the smaller institutions who, because the members of their committees of management are content with the incomplete analysis of figures they supply, and because the usefulness of a well-arranged system of accounts as an aid to economy has not occurred to them, imagine that the Uniform System is an unnecessary expenditure of time and trouble. It should hardly be necessary to point out that the Uniform System of Accounts was not wholly intended to satisfy the requirements of individual committees. It was devised in order that there might be an inter-hospital standard for the whole voluntary system, and this being so, any request for further details likely to prove generally helpful should merit the consideration of hospital workers.

#### Additional Details Required.

It is common knowledge that much of the correspondence which is carried on between the secretaries and committees of the various Institutions is occasioned by the want of detail which the published accounts display. At the moment, for instance, the small hospitals are beginning to consider the advisability of installing *x*-ray apparatus. An instance of this was given in The Hospital only two weeks ago. The small institutions, however, are hindered in their good intentions largely by two factors. One is a fear of the annual expense involved by the maintenance of the apparatus; the other is the difficulty experienced in finding out what the cost of installation and maintenance is. It seems unfortunate that a carefully compiled system of accounts should not disclose such an important section of expenditure. The fact is, of course, that the account-books contain the details which are massed under general headings in the statement which is published in the report.

The Uniform System involves a very close analysis of expenditure in all departments, and if it be properly carried out the analysis journal should reveal much valuable information which could quite easily be extracted.

#### How to Meet the Need.

Fuller details respecting important sections of expenditure need not involve any modification of the annual statement as at present published. The necessary information could be furnished by the issue of subsidiary statements or returns, as is done in the case of the washing return, and here it will be useful to note that the value of such returns depends upon the amplification of details given. The standard washing return would be much more valuable if the expenditure upon different classes of material used were given separately. For instance, inquiries are frequently made as to what is the average quantity of soap used for washing a certain number of pieces; this information cannot be extracted from the return because it is customary to include other items of expenditure under the same heading, and the quantities are not noted.

#### Suggestion to Hospital Workers.

The present article, therefore, takes this latest demand for information as a good instance of the need, not for additional calculation, but for further publication of detail. It is only a small matter, and at any rate should be allowed the chance of a verdict which discussion and criticism alone can give. For these the columns of "The Institutional Worker" are open, and if advantage be taken of the opportunity afforded by this section of The Hospital it will become clear in what departments fuller details are specially desired by hospital workers, and the first step will have been taken towards supplying the demand. It certainly seems as if details of the cost of upkeep, not merely of the *x*-ray department but also of the pathological department, would be worth rescuing from their present burial ground in the secretary's account-books.

### Subsidiary Organisations.

Local personal interest on which alone hospital finance, and indeed efficiency, depend cannot better be stimulated than by the extension of subsidiary organisations which have as an object the promotion of the general welfare of the institution, but which do not in any way interfere with the general administration. The usefulness of a ladies' committee in this respect is a happy example. There are so many ways in which a well-organised body of ladies can apply their energies to such work far more effectively than men; and provided, of course, that certain definite aims are kept in the foreground, women's meetings have less tendency to degenerate into a mere record of attendances and abstract discussions. The character of the work usually performed by ladies demands actual personal service by each member of the committee, especially in the case of a linen league or guild. The snowball system whereby certain of the members, able to devote a small annual sum to the work and a little spare time, undertake to secure from amongst their friends bands of assistants or associates of whom each one contributes a small subscription and performs an allotted share of work, brings into close contact with the institution all sections of the community. Their work does not end with the supply of every article of linen and clothing required in the hospital, which is generally the object of the league, and in itself is a very substantial service; consciously and unconsciously a system of propaganda is being carried on which brings an ever-increasing number of people into closer relations with the institution. The personal act of making or providing some necessary article for a patient stimulates an interest in the patient, and so the ball rolls on. And yet we are acquainted with more than one institution where such an organisation has been allowed to die. This is a disastrous mistake, for the voluntary basis of our hospitals, both financially and from the point of view of efficiency also, as explained above, depends ultimately on the personal ties which these organisations evoke.

### Questions for September.

No. 1. Annual Cleaning

**What must be the essential details involved in the annual cleaning and painting, and what is the best method of supervising the work? [Detailed information as to the type and relative cost of paints and enamels found by experience to be most suitable should be given, and any suggestion on the best way to make the supervision effective will be favourably regarded in selecting the replies.**

No. 2. X-Ray Apparatus.

**Give particulars of the X-ray apparatus in use at your institution, the names of makers and cost of installation State the number of each class of cases dealt with, and give a detailed account of the annual cost of maintenance**

RULES.

The following rules must be observed:—

1. Contributions must be written on one side of the paper. They must bear the name and address of the sender and be accompanied by coupon to be cut from the back cover (inside page) of the current issue of The Hospital. A pseudonym must be chosen if the name is not to be published.
2. They must be addressed to the Editor of The Hospital, 28 & 29 Southampton Street, Strand, London, W.C., so as to reach him before the end of the month, and must be marked in the left-hand corner "Facts and Figures."

**A minimum payment of Half a Guinea will be made for each published answer.**

### Any Use for an Addressograph?

We publish hereunder the opinion of another institutional worker upon this subject:

Unless the same names and addresses are constantly required, it is far easier and more economical for a hospital to have written envelopes; and this fact will be evident from the following description of the use of the machine. The addressograph is designed for reproducing addresses from metal plates, each of which has previously been embossed with an address. These plates are filed in light steel drawers, so that they may be ready for use at any time. To print the addresses the operator transfers the address plates direct from the drawer to the upright magazine of the addressograph. The empty drawer is then placed in the drawer-holder beneath the printing point of the machine to receive the plates in their original order after being printed. The machine is worked by a foot lever, a light forward swing of which prints an address, automatically passes the plate into the drawer, at the same time inking and bringing the next address plate into position for printing. Additional address plates can either be made with a graphotype or obtained from the Addressograph Co. if the number of addresses does not justify the purchase of this machine. On the whole, I think that if the number of subscribers to a hospital exceeded, say 4,000, it would be a saving of time to have an addressograph; but, on the other hand, a typed envelope has the tendency to be thrown into the waste-paper basket. An envelope nicely handwritten cannot be equalled.—"Nil Desperandum."

### THE EDITOR'S LETTER-BOX.

*THE SICK AND IN NEED.*

### Convalescent Letter Required.

We appeal on behalf of a poor man, who is urgently in need of a change and rest, for a subscriber's letter of recommendation to a convalescent institution; preferably the Metropolitan Convalescent Institution, Bexhill-on-Sea.

### Home of Rest for Nurse.

We suggest that you apply to the following institutions on behalf of the nurse who requires a rest: Merchant Taylors' Convalescent Home, Bognor, admission upon application to the Clerk, Merchant Taylors' Hall, Threadneedle Street, E.C.; The Claughton Convalescent Home, Walton-on-the-Naze, from 7s. to 25s. a week; Home of Rest, Shanklin, Isle of Wight, from 11s. a week, without a subscriber's letter; Home of Rest, Canterbury House, Alexandra Gardens, Folkestone, 15s. 6d. a week, and Princess Marie Louise House, Y.M.C.A., Worthing, 10s. 6d. a week.—E. W.

### Help Wanted for District Nurse.

A district nurse who has completely broken down in health with bad heart disease, and has been unable to work for the past year, is much in need of help pending her admission to a permanent home. She is only a little over thirty years of age, but there is no prospect of her continuing her nursing career, and she is entirely without means. She is a good needlewoman, and can do light work of this kind when well enough; at present she is able to get about and help herself, but she is quite unfit for regular employment. We appeal to our readers to co-operate with us in finding the needful help. Offers of assistance or suggestions will be gratefully welcomed, and should be addressed to V. H. B., 170.

*ACKNOWLEDGMENTS.*

**C. E. J.**—We regret to hear that you have not been successful. We suggest that you try St. Elizabeth's Home, 59 Mortimer Street, W., and Filston Home, 26 Bloomsbury Place, Brighton.

**V. H. B.**—Thank you for your letter; we are making further inquiries on behalf of the district nurse, and we are hopeful that some means of helping her, pending her admission to a suitable home, will be found. We ascertained that cases of chronic heart trouble are eligible for admission to the Putney Hospital, and we shall be glad to hear on what grounds your application has been refused.

*Communications have been received from the following, and enclosures forwarded: M. Aldridge, L. Starey, E. Coulter, Sister Arnold, H. Wood, M. E. A. Martin, M. J. Rumball, Miss Greenwood, Mrs. Ingle, and F. Warburton.*

*Communications from "Helpful" and F. H. are being attended to.*

**We desire to inform numerous correspondents that we are unable to forward letters from or recommend any but our Approved Homes.**

*MISCELLANEOUS.*

### National Insurance Problems.

1. Yes, your resident medical officer for the time being can be placed on *limited panel*, with a view to treating insured persons of the staff by special arrangement with the Insurance Committee for your area.

2. Paid members of a hospital staff are in general "employed persons within the meaning of the Act," and have to be insured. If they receive remuneration of greater value than £160 a year, they need not be insured, nor if it can be shown to the satisfaction of the Insurance Commissioners that the hospital employment is a subsidiary occupation. In certain circumstances it is possible for persons to obtain certificates of exemption.—S. S.

### Pension or Home on the Riviera.

We fear it is quite impossible to find a Pension with terms such as you propose—less than 3s. a day for everything, board, lodging, and attendance. The cheapest Pensions (and there are but very few of them) charge 5 francs a day, or £1 8s. 4d. a week. Try Miss Heale, 7 Avenue Riondet, Hyères, and Madame Chabert, 6 Rue Assalit, Nice, from 4½ francs. Pension Internationale, Borgio Verezzi, about the same terms, though sometimes you can get a slight reduction on a stay of a few weeks. Villa Corradi, Bordighera, from 5 lire—a lira is equivalent to a franc; this house is managed by French nuns, and is, therefore, pretty sure to be comfortable. The only inexpensive Home for invalid ladies that we know of is Villa Emily, San Remo, the terms are 25s. a week, and all particulars may be obtained from Miss Ferguson Corrachee, Tarland, Aberdeenshire. If you think of inquiring about the others, do so on a prepaid foreign return letter-card.—"Romana."

### Unbreakable Hot-Water Bottle.

An excellent hot-water bottle, which has been found very satisfactory for hospital use, can be obtained from Messrs. Jones Bros. and Co., of Wolverhampton. It is made of aluminium, and, being drawn from one piece of metal, it is jointless, so that there is no danger of its leaking. It is light, durable, and easily cleaned, though care must be taken that soda or potash are not brought into contact with it. The design originated at the Wolverhampton Hospital.—M. J.

**The Editor is prepared to make known without charge the needs of any who may be sick or in difficulties, and to guide them in making choice of Homes for treatment or convalescence. Reduced terms can often be arranged with Homes on the Approved List for those unable to pay full fees. No Home is included in the Approved List without medical and other testimony to its good management. Queries are specially invited from those who are engaged in any kind of philanthropical work.**

### Editor's Notices.

**Contributions:**

Contributions should be written, or preferably typed, on one side of the paper only, and all articles sent in are accepted upon the distinct understanding that they are forwarded to The Hospital only.

The Editor cannot undertake to return MSS. not used, but when a stamped directed envelope is enclosed the MSS. may be returned if a special request is made.

Accepted articles and paragraphs of news will be paid for after publication at the scale rate.

**Address:**

To prevent delay all contributions and letters on editorial business must be addressed exclusively to the Editor, "The Hospital," 29 Southampton Street, Strand, London, W.C. It is important that this regulation shall be strictly observed.

**Correspondence:**

Correspondence on all subjects is invited. The name and address of correspondents must be given as a guarantee of good faith, but not necessarily for publication.

**Special Articles:**

Special articles are invited, and questions, inquiries, and paragraphs upon all matters relating to the work, administration and management of general, special, mental, fever, cottage and convalescent hospitals, sanatoria, homes, institutions, societies and organisations for the treatment or care of the sick, injured and dependants of all classes. Every matter affecting the interests and welfare of the staff of all grades working in these institutions will receive special consideration.

**Administrative Medicine, Research and Items of News:**

Special terms will be paid for approved articles on subjects in this wide field by experts who have made some section of it their special study and interest. We wish to give prominence to every movement tending to advance accuracy of diagnosis, efficiency in treatment, and the development of modern methods to eradicate disease and promote the welfare of the suffering.

**A Bureau of Information:**

We invite inquiries and applications for information and help in providing for that numerous class of sufferers who require special care or house-room which they cannot obtain in their own homes or secure for themselves. We cannot, however, prescribe, or recommend practitioners

**Books for Review:**

Publishers are particularly requested to send advance proofs of any new books of importance whenever possible, as the Editor has made arrangements to publish immediate reviews on a new plan.

**Photographs, Plans, Blocks, and Illustrations:**

It is requested that wherever possible MSS. may be accompanied by illustrations in any of the above forms. The name of the sender and of the article to which it belongs should be written on the back of each photograph, drawing, or block for purposes of identification.

**Local Papers:**

Newspapers containing reports or news paragraphs should be marked and addressed to the Sub-Editor.

**The Coupon System:**

A coupon will be found at the bottom of the third inside page of the cover each week. This coupon must be attached to every question or inquiry to which an answer is desired in The Hospital.

### The Worker's Help.

Our purpose is to make this Supplement of The Hospital of the greatest possible service to every Institutional Worker. The term is employed in its widest sense, and includes all those individuals engaged in or associated with Charitable, Poor-Law, and Municipal Administration, as well as the Medical, Health, and Sanitary Services.

Although the section is primarily intended to help those seeking appointments, and numerous announcements of vacancies appear in its columns every week, it is felt there are also many other ways in which it can be of value. A number of pages have recently been added containing information which it is hoped will prove of assistance to many of our readers in their work, and in this you can help us and yourself by showing a lively interest in this section and offering such suggestions for its improvement as may occur to you.

### Manager's Notices.

**Letters relating to the publication, sale, and advertising departments of "The Hospital" must be addressed to The Manager, "The Hospital," The Hospital Building, 28 and 29 Southampton Street, London, W.C.**

**Advertisements:**

To ensure insertion, all advertisements for The Hospital must reach the Manager not later than Wednesday morning in each week. For scale of charges see pages xix and 4.

**Remittances:**

**It is especially requested that remittances be made by Cheque or Postal Order, and in halfpenny stamps only when the amount is under sixpence.**

**Subscriptions** may begin at any time, and are payable in advance. Cheques and Post-Office Orders should be crossed London County and Westminster Bank, Covent Garden Branch, and made payable to the Manager of The Hospital.

Rates of Subscription (including postage).

| *United Kingdom.* | | *Foreign and Colonial.* | |
|---|---|---|---|
| Three Months ... | ... 2s. 0d. | Three Months ... | ... 2s. 6d. |
| Six Months ... | ... 4s. 0d | Six Months ... | ... 5s. 0d. |
| Twelve Months ... | ... 6s. 6d | Twelve Months ... | ... 8s. 6d. |

*SUBSCRIPTION FORM.*

*Please send me* The Hospital *for..............months, commencing with your issue of.............................., for which please find enclosed............................*

*Name*.........................................................

*Address* ........................................................

........................................................

*Date*......................................

*To* .................................... *Newsagent.*

Or to

*The Manager,*
The Hospital,
The Hospital Building,
28/29 Southampton Street,
Strand, London, W.C.

